# Appearance of myocarditis lesions on spectral CT arterial acquisitions and correlation with edema on MRI

**DOI:** 10.1186/s41747-025-00613-x

**Published:** 2025-09-11

**Authors:** Sara Boccalini, Clara Fourrier, Salim Si-Mohamed, Eric Bonnefoy-Cudraz, Thomas Bochaton, Loic Boussel, Anna Vlachomitrou, Rafael Wiemker, Philippe Douek

**Affiliations:** 1 Department of Cardiovascular and Thoracic Radiology, Louis Pradel Hospital, Hospices Civils de Lyon, Bron, France; 2 University Lyon, INSA-Lyon, University Claude Bernard Lyon 1, UJM-Saint Etienne, CNRS, Inserm, Villeurbanne, France; 3 Department of Cardiology, Louis Pradel Hospital, Hospices Civils de Lyon, Bron, France; 4 University of Lyon, INSA-Lyon, University Claude Bernard Lyon 1, Lyon, France; 5 Philips Healthcare, Suresnes, France; 6 Philips Innovative Technologies, Hamburg, Germany

**Keywords:** Coronary vessels, Edema (cardiac), Magnetic resonance imaging, Myocarditis, Tomography (X-ray computed)

## Abstract

**Background:**

Spectral computed tomography (CT) late-enhancement (LE) acquisitions can help detect myocarditis. An arterial acquisition is often performed for coronary artery analysis. However, little is known about the appearance of myocarditis on the arterial phase. We investigated the appearance of myocarditis on arterial acquisitions of cardiac spectral CT, and its relationship to LE and edema.

**Materials and methods:**

Forty-seven cardiac spectral CTs performed in patients with magnetic resonance imaging (MRI)-confirmed myocarditis were retrospectively assessed. Three myocardial attenuation/enhancement patterns were visually identified and segmented on both arterial and LE acquisitions: hypodense-arterial + normal-LE (HypoArt–NorLE); normal-arterial + hyperdense-LE (NorArt–HyperLE); and hypodense-arterial + hyperdense-late (HypoArt–HyperLE). Characteristics of conventional and spectral images were calculated for all patterns and for remote myocardium. Values of HypoArt–HyperLE lesions were compared in the groups with and without edema on MRI, as assessed with T2 mapping (available for 25 patients).

**Results:**

We found 173 lesions, 46 (26%) HypoArt–NorLE, 54 (31%) NorArt–HyperLE, and 73 (42%) HypoArt–HyperLE. On the arterial phase, HypoArt–HyperLE were more hypodense (*p* < 0.001) and had less iodine (0.23 mg/mL less; *p* < 0.001) than RM. On LE, both HypoArt–HyperLE and NorArt–HyperLE were more hyperdense and contained more iodine than the remote myocardium (all *p* < 0.001). HypoArt–HyperLE lesions were more hypodense and contained less iodine on the arterial phase in patients with edema on MRI as compared to those without (all *p* < 0.001).

**Conclusion:**

Most myocarditis lesions detectable with spectral CT are visible on both arterial and LE acquisitions. These lesions appeared to be more pronounced on the arterial phase in patients with edema on MRI.

**Relevance statement:**

Spectral CT arterial acquisition performed for the differential diagnosis of acute myocardial pathologies in many cases can depict myocarditis lesions as epicardial hypodense areas, most likely related to the presence of edema.

**Key Points:**

- Data from spectral CT shows that most myocarditis lesions appear as hypodense on the arterial phase, matching the epicardial LE zones.
- A minority of myocarditis lesions appear as epicardial LE areas without anomalies of attenuation on the arterial phase.
- Hypodense myocardial areas are correlated to the presence of edema on MRI, suggesting they are due to the same phenomenon.

**Graphical Abstract:**

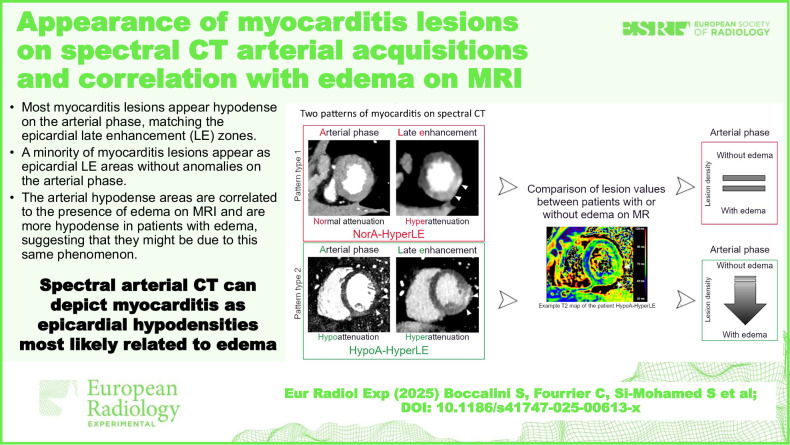

## Background

Acute myocarditis (AM) can pose a diagnostic challenge due to the wide range of possible clinical presentations that can mimic other cardiac pathologies, such as myocardial infarction [1, 2]. Early diagnosis and treatment are essential to prevent disease progression and long-term complications, including dilated cardiomyopathy [2, 3]. Traditionally, the diagnosis of AM is suspected based on clinical history, biological parameters, and electrocardiographic alterations, while myocardial biopsy remains the gold standard. However, nowadays magnetic resonance imaging (MRI), plays a central role in the diagnosis of AM, especially in stable patients [2, 4, 5]. Since 2018, the modified Lake Louise criteria [6] constitute the basis for the diagnosis of myocarditis, with two main criteria to be met: the presence of myocardial edema and non-ischemic myocardial T1 anomalies, including late-enhancement (LE) areas. Nevertheless, cardiac MRI is not always readily available, if available at all, and might be challenging to perform in acute scenarios.

Recently, computed tomography (CT) at low kV imaging has shown promising results for the differential diagnosis of AM and myocardial infarction [7]. Nevertheless, these images are hampered by high noise and low contrast resolution. Spectral CT can help overcome these limitations by improving contrast resolution, allowing for the detection of subtle changes in tissue density that may not be visible on conventional CT scans [8, 9]. In addition, spectral CT imaging allows for analysis of the coronary arteries, which is not possible in clinical routine with MRI, resulting in a more thorough assessment of the heart. Therefore, spectral CT imaging could potentially improve the clinical management of patients with AM, by providing a prompt diagnosis, particularly in cases where MRI is not readily available or feasible [10]. However, the diagnostic performance of spectral CT imaging for AM has not been fully established, and several aspects remain to be investigated. For instance, most spectral CT imaging studies focused on the detection of late iodine enhancement (LIE) while less attention has been given to myocardial edema, one of the two major criteria for MRI [7, 11, 12].

Therefore, the aim of our study was to evaluate the spectral characteristics of different patterns of AM lesions on arterial and late enhancement acquisitions as obtained with spectral CT and to compare them to the presence of edema on cardiac MRI.

## Materials and methods

We conducted a retrospective, observational, monocentric study in a tertiary University Hospital. The radiological database of the hospital was searched for patients who underwent a cardiac spectral CT for a suspicion of myocarditis between June 2017 and April 2020. The need for informed consent was waived by the local ethical committee due to the retrospective nature of the study (IRB N 24-5243).

### Population

**Fig. 1:**
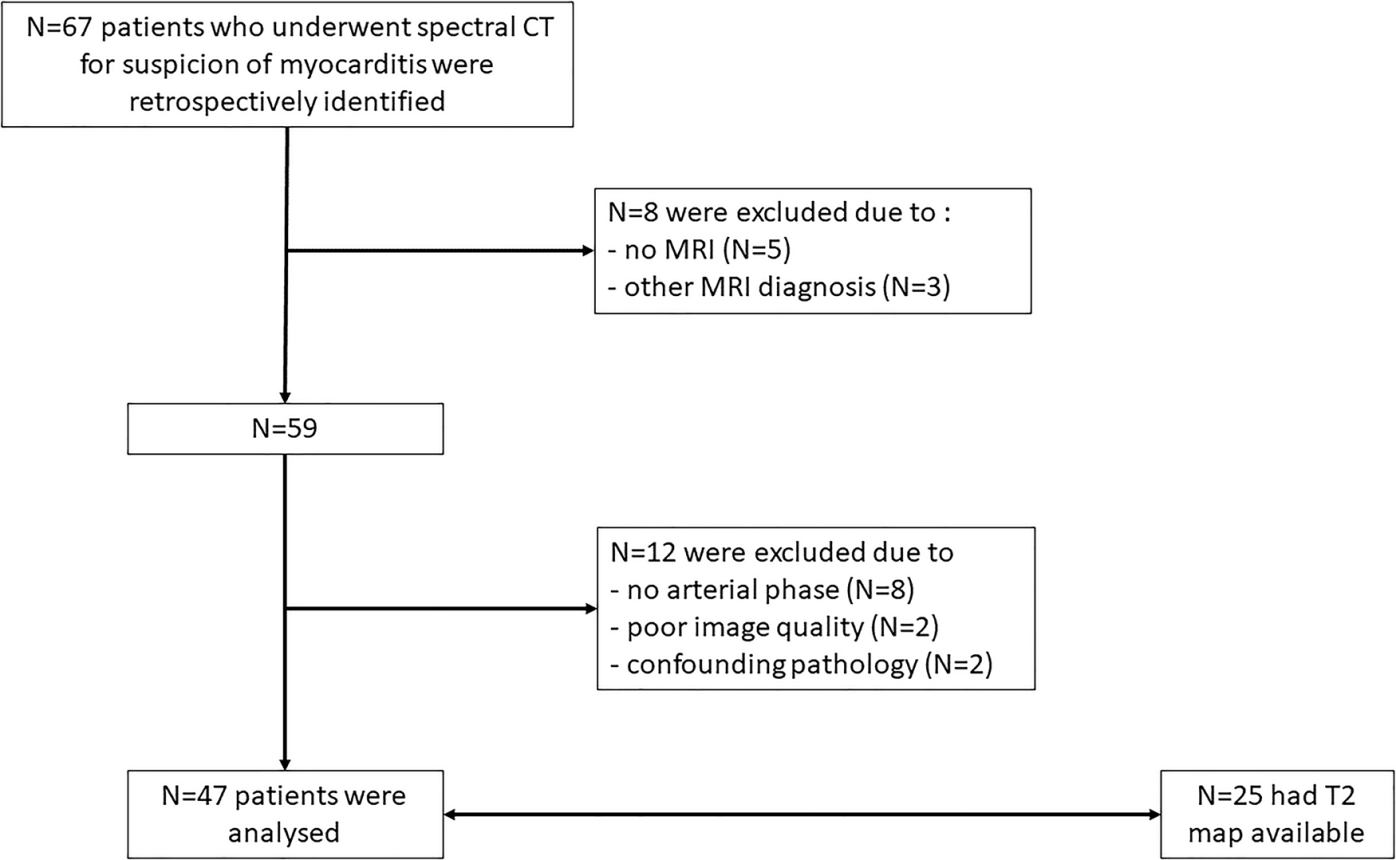
Flowchart of the study

The analysis of the radiological database allowed identification of 67 consecutive patients who underwent a cardiac spectral CT exam for the suspicion of myocarditis because of acute chest pain and elevated troponin values. These exams were performed shortly after the admission of the patient to our center to exclude the presence of coronary artery disease, as well as for the differential diagnosis of acute myocardial lesions. Of these, only those who had had a subsequent cardiac MRI (*n* = 62) confirming the diagnosis of AM (*n* = 59), according to the criteria in use at the time of the clinical exam, that is the so called Lake Louis Criteria or the modified Lake Louis criteria [6, 10], were further examined. CT scans for which the spectral arterial images were not available (*n* = 12) or the image quality was judged insufficient (*n* = 2), as well as patients who had confounding pathologies (*n* = 2), were excluded. The flowchart of the study with inclusion and exclusion criteria is shown in Fig. 1. In total, the CT scans of 47 patients were included and analyzed. In one patient who had both a myocardial infarction and MA, only the lesions of myocarditis were analyzed, while the infarcted area was excluded from further analysis.

### Spectral CT acquisitions and reconstructions

All examinations have been performed on a dual-layer dual-energy CT (IQon; Philips) and included a first-pass coronary artery acquisition and a late acquisition, 10 min after injection. If indicated and in the absence of contraindications, intravenous beta-blockers and sublingual spray of nitroglycerin were administered prior to the arterial injection (esmolol chlorohydrate, Esmocard, Orpha Devel Handels Vertriebs GMBH; and Natispray, Teofarma SRL). The injection protocol included a total of 1.2 mL/kg of iodinated contrast media (Iomeron 400 mg/mL, Bracco), of which the first 50–60 mL, depending on the size of the patient, were administered for the arterial phase and the rest just after it, 2–3 min after the beginning of the first injection. The timing of the arterial acquisition was established with bolus tracking by placing a region of interest in the proximal descending aorta and using a threshold of 130 HU. The second acquisition started 10 min after the beginning of the first injection of contrast media. An average of 77.7 ± 18 mL of contrast agent was injected. Acquisition and reconstruction parameters are reported in Table 1.

**Table 1:** Acquisition and reconstruction parameters of CT scans

	Arterial phase	Late phase
Acquisition parameters		
Collimation [mm]	64 × 0.625	64 × 0.625
Tube voltage [kVp]	120	120
Tube current [mAs]	255	103
Rotation time [s]	0.27	0.27
Pitch	0.16	0.2
Dose modulation	DoseRight	DoseRight
Reconstruction parameters		
Phase of the cardiac cycle [%]	78	75
Field of view [mm]	500	500
Thickness [mm]	2.5	2.5
Kernel	Cardiac standard (CB)	Cardiac smooth (CA)
Iterative reconstruction	iDose^4^ level 4	iDose^4^ level 7
Radiation dose		
Dose-length product [mGy/cm]	421.4 ± 202	130.5 ± 54.4
CT dose index [mGy]	28.4 ± 50.5	16.7 ± 2.5

*CT* Computed tomography

### Spectral CT image analysis

The analysis of the myocardium was carried out with research software (Philips Research), which allows for simultaneous annotations and spectral data extraction on two different spectral CT acquisitions (*i.e*., arterial and LE).

**Fig. 2:**
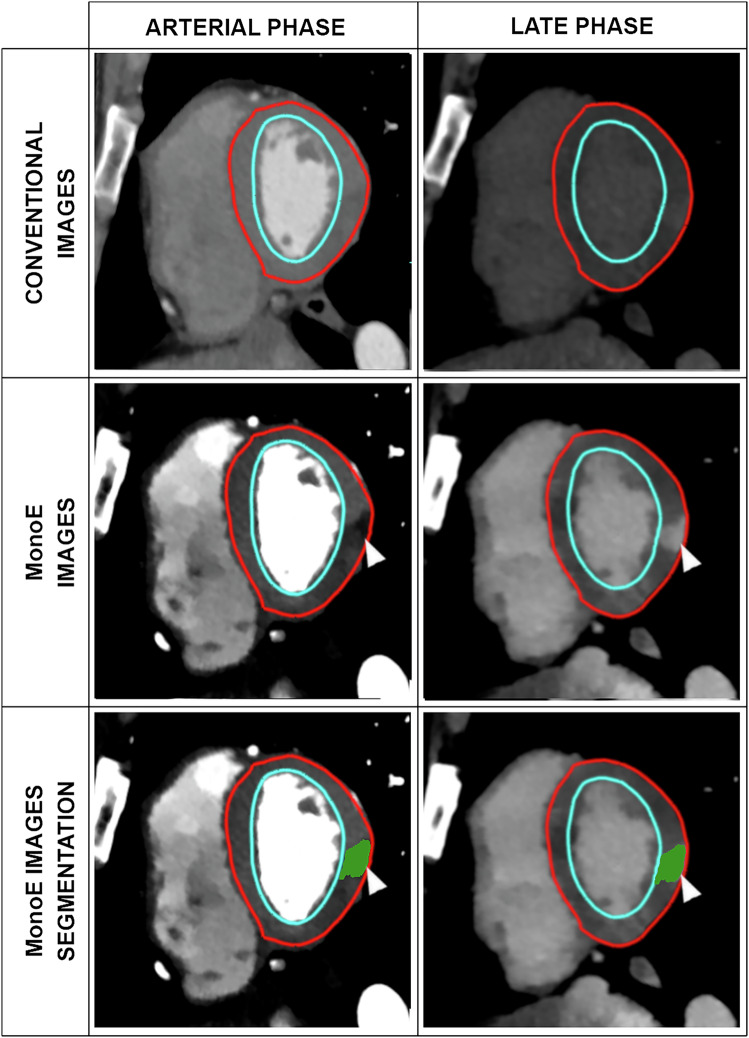
Functioning of our research tool. On the first line, conventional images with myocardial contouring (the blue line represents the endocardium, the red line represents the epicardium). On the second line, the corresponding spectral images (40 keV monoenergetic images) show an example of HypoArt–NorLE (black arrowheads) and HypoArt–HyperLE (white arrowheads) areas. On the third line, the same images with color-coded annotations (HypoArt–NorLE in blue and HypoArt–HyperLE in green on both arterial and LE acquisitions) after automatic transposition on the two acquisitions. HypoArt–HyperLE, Hypodense at arterial phase and hyperdense at late enhancement phase; HypoArt-Nor, Hypodense at arterial phase and isodense at late enhancement phase; NorArt–HyperLE, Isodense at arterial phase and hyperdense at late enhancement phase

First, the entire myocardium of the left ventricle was semiautomatically and independently segmented for the two acquisitions, arterial and LE. After automatic model adaptation of the endocardium and epicardium, inaccurate contouring was manually corrected (Fig. 2). Thereafter, the software automatically divided each of the 17 American Heart Association (AHA) segments in 4 × 3 × 3 subvolumes (4 in the endocardial to epicardial direction, 3 in the septum to lateral direction, and 3 in the basal to apex direction) that were the basic unit for manual lesion annotations. A radiology resident (C.F.), with 6 months of experience in cardiovascular imaging corrected all contours and defined the lesions of AM. A radiologist (S.B.) with 11 years of experience in cardiovascular imaging reviewed and corrected, if necessary, all contours and annotations. The readers were free to use conventional or spectral images for the identification and characterization of myocardial lesions.

**Fig. 3:**
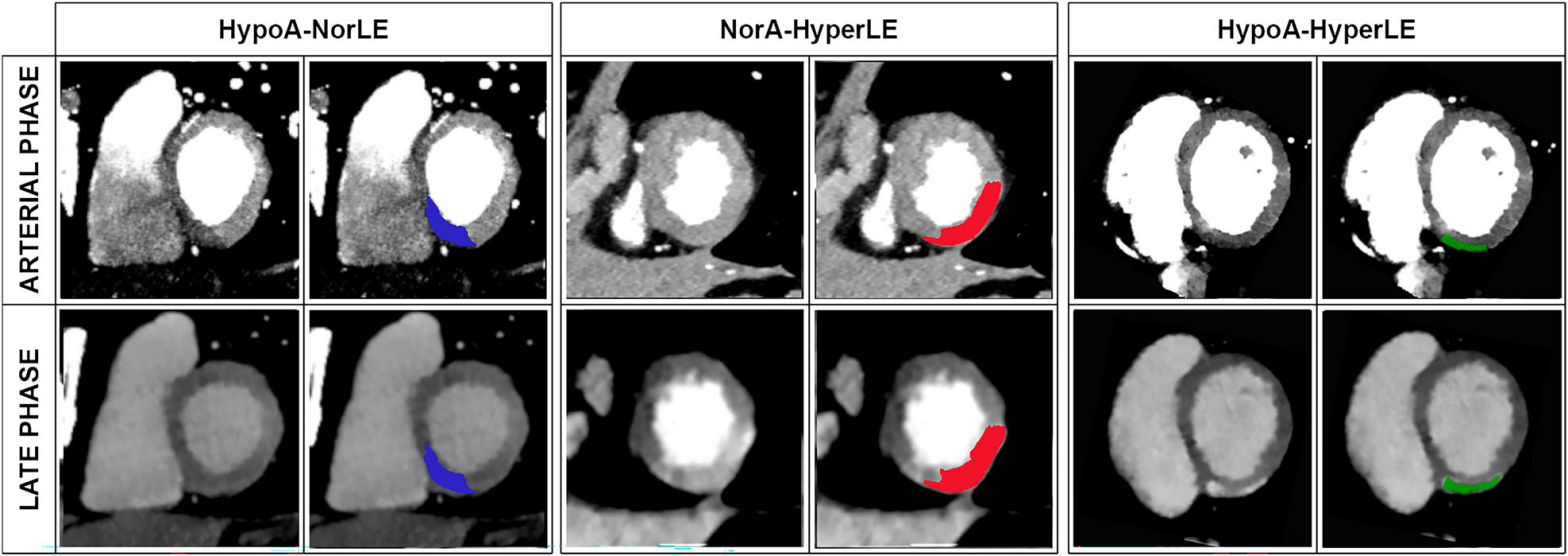
The three different types of lesions are shown with color-coding on arterial and matching late phase on spectral CT. The blue color represents lesions that are hypodense on the arterial phase and are isodense on the late phase (HypoArt–NorLE). The red color represents the lesions isodense on the arterial phase and hyperdense at the late phase (NorArt–HyperLE). In green, the lesions that were hypodense on the arterial phase and hyperdense on the late phase (HypoArt–HyperLE)

For the purpose of this study, three types of different attenuation/enhancement patterns were defined as follows (Figs. 2 and 3):

- NorArt–HyperLE: normal arterial + hyperdense LE;
- HypoArt–NorLE: hypodense arterial + isodense LE;
- HypoArt–HyperLE: hypodense arterial + hyperdense LE.

All lesions defined on one acquisition were automatically transposed to the same location on the other acquisition (Fig. 2). This was done by a three-dimensional landmark-based interpolation of the anchoring grid of the two automatically adapted AHA models. Thereafter, manual selection of any AHA-subsegment was applied automatically to both scans. The remote myocardium (RM) considered as visually normal was calculated and analyzed by subtraction by the software (RM = entire myocardium − lesions).

For each lesion, the position relative to the endocardium and epicardium was automatically noted. For the objective of this study, lesions affecting ≤ 50% of the thickness of the myocardium starting from the epicardium were considered epicardial, and lesions > 50% of the thickness were considered transmural.

Then, the software extracted and allowed exporting conventional and spectral data of the RM and of the myocarditis lesions. Spectral data included virtual monoenergetic images at low energy (40 keV and 50 keV) and iodine density maps [12–14].

### MRI acquisitions and comparison of arterial CT findings *versus* edema on MRI

Examinations have been performed on a 1.5-T scanner (Ingenia, Philips). For 25 patients, the MRI protocol included T2 mapping sequences performed on three short-axis planes (basal, mid-ventricular, and apical). Only MRIs performed within 4 days after CT were considered for further analysis. The highest value of T2 mapping per patient was calculated as follows: first, a color-coded representation of the three T2 maps images was visually inspected; second, for each slice, a free-hand region of interest of at least 1 cm^2^ of surface was traced were the values were higher; finally, the highest of the three values was used for further analysis. A T2 map value of ≥ 60 ms was considered positive for the presence of edema in accordance with clinical routine evaluation in our department. The presence or not of edema on MRI was compared to the presence or not of HypoArt–HyperLE areas on the spectral CT examinations. In addition, values of attenuation and iodine concentration of lesions on CT acquisitions were compared between patients without and with edema on MRI.

### Statistical analysis

Statistical analysis was carried out with SPSS, version 21 (IBM, Massachusetts, USA). Categorical variables are presented as frequencies and percentages. The χ^2^ test and the McNemar test were employed to analyze differences in categorical variables. For continuous variables, normality was assessed with the Shapiro–Wilk test and QQ-plots. Friedman test and Kruskal–Wallis test with Dunn–Bonferroni *post hoc* test were employed to compare multiple groups of continuous variables. The Wilcoxon test was employed to test the differences between patients without and with edema. The Pearson or the Spearman test was used to assess correlations.

## Results

### Characteristics of the patients

The characteristics of the 47 included patients are detailed in Table 2.

**Table 2:** Patients’ characteristics (*n* = 47)

Patient characteristics	
Age [years]	29.9 ± 10.9
Sex	39 (83%) men
Height [cm]^∗^	175 ± 50
Weight [kg]^∗∗^	77 ± 17
Body mass index^∗^	25 ± 8
Cardiovascular risk factors	
Family history	6 (13%)
Smoke	18 (38%)
Cannabis consumption (smoking)	3 (6%)
Diabetes	1 (2%)
Arterial hypertension	1 (2%)
Previously known cardiac disease	
Left ventricular hypertrophy	1 (2%)
Dilated cardiomyopathy	1 (2%)
Symptoms	
Chest pain	47 (100%)
Onset of chest pain [days before CT]	1.9 ± 2.2
Palpitations	4 (9%)
Symptoms typical of pericarditis	9 (19%)
Symptoms and signs of viral infection^∗∗∗^	
Fever	8 (17%)
Diarrhea	5 (7%)
Nausea and/or vomiting	0
Both diarrhea and nausea and/or vomiting	5 (11%)
Angina	13 (28%)
Generic symptoms of viral infection	4 (9%)
Biological parameters	
High sensitivity troponins [ng/L]	7004 (9454)
C-reactive protein [ng/L]	28 (83)
Left ventricle ejection fraction [%]	57 ± 11
Electrocardiogram	
Normal	5 (11%)
Anomalies of the segment ST	27 (57%)
Anomalies of the segment PR	6 (13%)
Anomalies of the T wave	15 (32%)
Right bundle block	4 (9%) (3 incomplete)
Arrhythmia	3 (6%)
Coronary artery plaques on CT	3 (6%)
Stenosis 1–24%	2 (4%)
Stenosis 25–49%	1 (2%)
Invasive coronary angiography	6 (13%)
Occlusion	0
Significant stenosis	0
Complications	
None	45 (96%)
Dilated cardiomyopathy	1 (2%)
Arrhythmia	1 (2%)

Ordinal data are reported as mean ± standard deviation or median (interquartile range, expressed as Q3-Q1), depending on the distribution

*CT* computed tomography

^∗^ Data available for 43 patients

^∗∗^ Data available for 46 patients

^∗∗∗^ In the 10 days prior to admission

The etiology of AM was attributed to a viral infection in the vast majority of cases (*n* = 46, 98%), although the virus in question could not be established except for one patient who tested positive for Severe Acute Respiratory Syndrome Coronavirus 2. For one patient, the etiology was a bacterial infection (type B *Neisseria meningitidis*).

For all but two patients, the episode of chest pain with elevated troponins investigated in this study was the first one. The other two patients had already had two episodes of myopericarditis each. One of these patients and four others presented again with chest pain and troponin elevation in the years after (patients’ files searched in March 2025). Further investigations allowed us to find β-2-glycoprotein antibodies in one patient and a GATA binding protein 5 mutation (where GATA stands for the literal arrangement of nucleotides) in another patient. In both cases, it remains unclear if these anomalies were the direct causative effect of the episode of myocarditis. For all other cases, the putative cause remains viral.

Six patients (13%) were administered intravenous beta-blockers and sublingual nitrates immediately prior to the CT scan. The average time between the spectral CT and MRI examinations was 27 ± 62 days (mean ± standard deviation), with 29 (62%) MRI examinations performed in the week following the CT.

### General characteristics of the lesions

In total, 145 lesions were found, an average of 3.01 ± 1.99 (range 0–8) lesions per patient; 26 lesions presented more than one attenuation pattern, in all cases an association of HypoArt–HyperLE and NorArt–HyperLE areas, to which in two cases also a HypoArt–NorLE was added (Table 3). Therefore, 173 total areas with different attenuation patterns were identified. The most common pattern was HypoArt–HyperLE, accounting for 73 (42%) of pathological areas. The second most common pattern was NorArt–HyperLE (54 areas, 31%), followed by HypoArt–NorLE (46 areas, 26%). One example per type of lesion is presented in Fig. 3. The frequencies and data mentioned below are presented per area of attenuation pattern unless specified otherwise.

**Table 3:** Type and numbers of different lesions, and values of different types of attenuation areas on conventional and spectral images in the arterial and late enhancement phase

	HypoArt–NorLE	*p*-value	NorArt–HyperLE	*p*-value	HypoArt–HyperLE	*p*-value	Remote myocardium
Number of areas	46 (26%)		54 (31%)		73 (42%)		
Volume [number of subsegments]	157		227		486		
Arterial phase							
Attenuation							
Conventional images [HU]	71 (17)	1.000	70 (22)	0.139	61 (24)	**<** **0.001**	74 (9)
MonoE 40 keV [HU]	123 (49)	**<** **0.001**	147 (47)	1.000	124 (47)	**<** **0.001**	146 (32)
MonoE 50 keV [HU]	95 (30)	**<** **0.001**	108 (34)	1.000	93 (33)	**<** **0.001**	108 (17)
Iodine concentration [mg/mL]	0.92 (1.0)	**<** **0.001**	1.23 (1.0)	1.000	1.0 (0)	**0.001**	1.23 0)
Late enhancement phase							
Attenuation							
Conventional images [HU]	68 (8)	0.989	77 (21)	**<** **0.001**	76 (10)	**<** **0.001**	69 (8)
MonoE 40 keV [HU]	129 (37)	0.989	165 (69)	**<** **0.001**	163 (29)	**<** **0.001**	134 (28)
MonoE 50 keV [HU]	98 (23)	0.989	120 (45)	**<** **0.001**	118 (18)	**<** **0.001**	101 (19)
Iodine concentration [mg/mL]	1.03 (1.0)	1.000	1.47 (1.0)	**<** **0.001**	1.44 (0)	**<** **0.001**	1.09 (0)

Categorical data are reported as: number (percentage). Ordinal data are reported as mean ± standard deviation or median (interquartile range, expressed as Q3-Q1) depending on the distribution. The *p*-values indicate differences compared to the remote myocardium

*HypoArt–HyperLE* Hypodense at arterial phase and hyperdense at late enhancement phase, *HypoArt-Nor* Hypodense at arterial phase and isodense at late enhancement phase, *NorArt–HyperLE* Isodense at arterial phase and hyperdense at late enhancement phase

The bold values indicate statistically significant differences in both tables

**Fig. 4:**
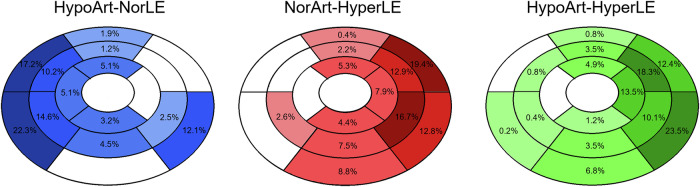
Bull’s eyes showing the percentage of the three types of lesions localized in the AHA 17 segments of the myocardium. HypoArt–HyperLE, Hypodense at arterial phase and hyperdense at late enhancement phase; HypoArt-Nor, Hypodense at arterial phase and isodense at late enhancement phase; NorArt–HyperLE, Isodense at arterial phase and hyperdense at late enhancement phase

The vast majority of lesions involved the epicardial layer (164, 95%). Of these, 37 had a transmural extension. The 9 lesions that did not involve the epicardial layer were mainly HypoArt–NorLE areas (6, 3%). Localization of the areas with different attenuation patterns in the 17 AHA is presented in Fig. 4.

### Spectral CT characteristics of the lesions

**Fig. 5:**
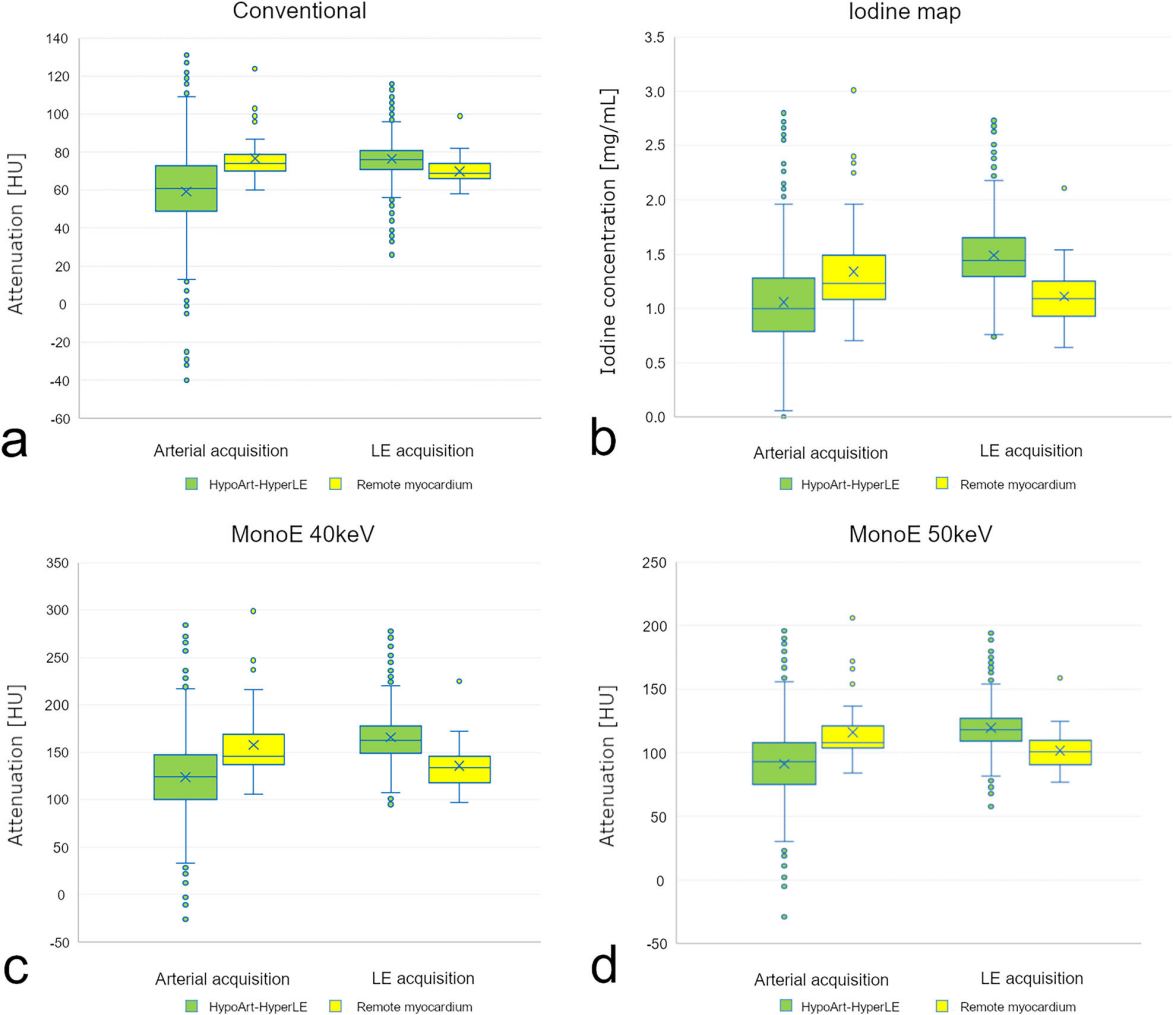
Boxplot graphs showing the attenuation on conventional (**a**), monoenergetic 40-keV (**c**), monoenergetic 50-keV (**d**) images, and iodine concentration (**b**) values of HypoArt–HyperLE lesions (in green) compared to the remote myocardium (in yellow), on both arterial and late enhancement acquisitions. HypoArt–HyperLE, Hypodense at arterial phase and hyperdense at late enhancement phase; HypoArt-Nor, Hypodense at arterial phase and isodense at late enhancement phase; NorArt–HyperLE, Isodense at arterial phase and hyperdense at late enhancement phase

Values of different types of attenuation areas on conventional and spectral images are reported in Table 3. Characteristics of the HypoArt–HyperLE areas as compared to the RM are shown in Fig. 5.

#### Differences between the lesions and the remote myocardium on the arterial phase

On conventional images, only HypoArt–HyperLE areas were significantly different as compared to RM (*p* < 0.001). In addition, HypoArt–HyperLE areas were significantly more hypodense than other lesions (all *p* < 0.001). On monoenergetic images at 40 keV, HypoArt–NorLE and HypoArt–HyperLE lesions were more hypodense than the RM (all *p* < 0.001) and had similar densities as compared to each other (*p* = 1.000). The difference between HypoArt–HyperLE *versus* RM was more evident on 40 keV-monoenergetic than on conventional images (22 HU *versus* 13 HU). The same results were found for 50 keV-monoenergetic and for iodine maps, with 0.23 mg/mL of difference in iodine concentration between HypoArt–HyperLE and RM. NorArt–HyperLE lesions did not show any significant difference as compared to the RM on any of the images (all *p* ≥ 0.050).

#### Differences between the lesions and the remote enhancement on the LE phase

Both NorArt–HyperLE and HypoArt–HyperLE lesions were more hyperdense than the RM on conventional, monoenergetic 40 kev, monoenergetic 50 keV images and contained more iodine on iodine maps (all *p* < 0.001). HypoArt–HyperLE lesions contained 0.35 mg/mL more iodine than the RM, and NorArt–HyperLE lesions 0.38 mg/mL more. There was no statistically significant difference between HypoArt–HyperLE and NorArt–HyperLE lesions on conventional nor on any of the spectral images (all *p* ≥ 0.943). HypoArt–NorLE lesions did not show any significant difference as compared to the RM on any type of images (all *p* ≥ 0.989).

### Comparison of lesions visible on late enhancement spectral CT and MRI

For five patients, no lesions were visible on the late enhancement acquisition of spectral CT. Of these, four patients had no late gadolinium enhancement (LGE) either while one patient had one epicardial lesion on MRI.

In all other cases, all the LIE lesions that were visible on spectral CT were also visible on MRI, with the exception of one lesion of segment 12 that was not visible on the MRI. However, in this case, the MRI was performed almost one year after the CT (334 days).

### Comparison of lesion characteristics on spectral CT and edema on MRI

**Fig. 6:**
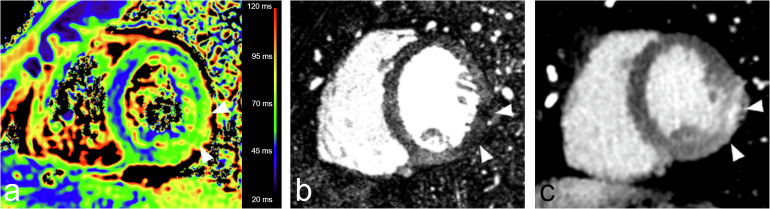
A 23-year-old man presenting to the emergency department with chest pain, a troponin level peak at 16,772 ng/mL, and a low risk of coronary artery disease. CT was performed on arrival, and magnetic resonance imaging was performed the following day. Increased values of T2 in the inferolateral wall of the left ventricle (**a**) corresponded to a HypoArt–HyperLE lesion: hypodensity on the arterial phase (arrowheads in **b**) and corresponding epicardial hyperdensity (arrowheads in **c**) on the late phase on monoenergetic images at 40 keV. The final diagnosis was of viral AM. HypoArt–HyperLE, Hypodense at arterial phase and hyperdense at late enhancement phase; HypoArt–Nor, Hypodense at arterial phase and isodense at late enhancement phase; NorArt–HyperLE, Isodense at arterial phase and hyperdense at late enhancement phase

In 25 patients, T2 mapping was performed and available on the picture archiving and communication system. In one case artifacts were too pronounced to allow for analysis. In 5 cases, the delay between the CT and the MRI examinations was > 4 days. Among the remaining 18 patients, 8 underwent the MRI the same day or the day after the spectral CT. The prevalence of edema on MRI (14 patients) and HypoArt–HyperLE lesions (20 patients) was similar (*p* = 0.625 and *p* = 0.155 for the McNemar and χ^2^ test, respectively). One example of a patient showing a HypoArt–HyperLE lesion of the lateral wall perfectly matching with an area of edema on T2 mapping is shown in Fig. 6.

In patients who had positive T2 mapping, HypoArt–HyperLE lesions had bigger volume, they were more hypodense on the arterial phase on monoenergetic spectral images (with a difference of 24 HU on 40 keV-monoenergetic) and contained less iodine (0.26 mg/mL of difference, about 20% less) than in patient who did not have edema on MRI (all *p* < 0.001). In the late enhancement phase, HypoArt–HyperLE lesions had slightly lower attenuation values and similar iodine concentration in patients with edema on MRI (*p* = 0.013 at 50 keV and *p* = 0.097, respectively) (Table 4).

**Table 4:** Comparison between HypoArt–HyperLE lesions in patients without and with edema on magnetic resonance imaging

HypoArt–HyperLE	Patients without edema on MRI (*n* = 5)	Patients with edema on MRI (*n* = 13)	*p*-value
Volume [mm^3^]	161 (64)	207 (139)	**<** **0.001**
Arterial phase			
Attenuation			
Conventional [HU]	66 (22)	63 (24)	0.071
Monoenergetic 40 keV [HU]	149 (30)	125 (40)	**<** **0.001**
Monoenergetic 50 keV [HU]	109 (23)	93 (31)	**<** **0.001**
Iodine concentration [mg/mL]	1.27 (0)	1.01 (0)	**<** **0.001**
Late enhancement phase			
Attenuation			
Conventional [HU]	80 (7)	76 (11)	**<** **0.001**
Monoenergetic 40 keV [HU]	175 (19)	169 (34)	0.053
Monoenergetic 50 keV [HU]	126 (12)	121 (21)	**0.017**
Iodine concentration [mg/mL]	1.6 (0)	1.53 (0)	0.433

Data are reported as median (interquartile range, expressed as Q3-Q1)

*HypoArt–HyperLE* Hypodense at arterial phase and hyperdense at late enhancement phase, *MRI* Magnetic resonance imaging

The bold values indicate statistically significant differences in both tables

NorArt–HyperLE lesions had similar attenuation and iodine content on the arterial acquisition in patients with or without T2 map positive values (*p* = 0.508 at 50 keV and *p* = 0.766, respectively). In the late enhancement phase, patients with edema had NorArt–HyperLE lesions that were denser (21 HU of difference on 40-keV monoenergetic; *p* = 0.014) and with more iodine (1.73 (interquartile Q3-Q1 = 1) *versus* 1.46 (interquartile Q3-Q1 = 1) mg/mL; *p* = 0.004) than in patients without edema.

RM had the same attenuation and the same iodine concentration on both arterial and late enhancement acquisitions (all *p* ≥ 0.053).

## Discussion

In our study, we showed that with cardiac spectral CT, epicardial LE lesions, which in the majority of cases are indicative of AM, are also visible on the arterial phase as areas of hypodensity and reduced iodine concentration (HypoArt–HyperLE lesions). In the LE phase, these lesions showed similar iodine content and attenuation as compared to areas of LE that were visible exclusively on the late acquisition (NorArt–HyperLE). Furthermore, only HypoArt–HyperLE lesions (and not NorArt–HyperLE nor RM) were significantly more hypodense on the arterial phase when T2 mapping was elevated, which suggests that hypodensity on the arterial phase corresponds to edema.

The main limitation of cardiac CT for myocardial assessment is the low contrast resolution of soft tissues, with the exception of fatty scars that can be detected on CT scans even without the need to inject and even if of small size [15]. However, this limitation can be overcome by spectral CT. In fact, spectral CT has proven its capability to increase the detection rate of LIE. For instance, in a recent article, spectral CT exhibited a 100% sensitivity in detecting LIE due to myocarditis, as compared to LGE [11].

The second limitation to the use of CT is that, for MRI, LGE is only one of the different parameters to be considered to reach the final diagnosis, including for AM. In fact, the modified Lake Louise criteria of 2018 [6] have even further highlighted the importance of the detection of myocardial edema as compared to the previous ones [10], designating it as one of the two major criteria for the diagnosis of AM. Edema identification is essential in non-viral myocarditis, where late enhancement is often absent, as demonstrated by a growing body of evidence [16, 17]. Two recent studies showed that up to 18% of patients with myocarditis induced by immune checkpoint inhibitor treatment for cancer had no LGE in MRI [16, 17]. MRI detectable anomalies in these patients include edema, increased values of T1 mapping, and elevated extracellular volume (ECV). So far, the latter is the only one that has been studied also with CT for patients with myocarditis [12, 18]. On the contrary, data about the other parameters is lacking. In addition to its relevance for diagnosis, elevated T2 map values are associated with increased risks of complications [10, 19]. Given the ensuing importance of edema detection, for both diagnosis and prognosis, the possibility of detecting it also on CT seems important for its broader application in clinical routine.

Our data shows that most “typical” myocarditis lesions (that is, lesions visible on the late enhancement phase as hyperdense areas) have a corresponding hypodensity on the arterial phase. The most likely explanation for this phenomenon is that the presence of edema in the area affected impedes the uptake of contrast media during this very early acquisition, as already suggested by some authors on isolated cases [20]. Our comparison to MRI data seems to confirm this hypothesis. Surely, we did not perform an area-to-area comparison, but this would be difficult for many reasons, including the different nature of MRI and CT, in particular, the fact that with MRI, only some slices are routinely performed for T2 mapping. In addition, the time elapsed between the exams is an important parameter to be taken into account, as it might potentially hamper the results of a direct comparison due to the edema reduction over time. Therefore, we tried to minimize the impact of the time elapsed between the two exams by considering only those performed within a few days.

Another interesting opportunity for further advancements is to exploit material decomposition and spectral properties in order to directly confirm the presence of edema [8]. This approach could yield significant advancements, in that it would also allow for the quantification of water, possibly allowing for the detection of diffuse edema as well. In fact, our analysis was limited to focal areas of hypodensity. More in particular, we simultaneously analyzed corresponding zones of the myocardium on the two acquisitions. During our analysis, we did not find this to be a limitation since all visually detectable arterial hypodensity corresponded exactly to the area of LIE in our patients. Nevertheless, this means that potential diffuse hypodensities were not taken into account. Moreover, the iodine concentration in healthy myocardium depends on factors such as the protocol of contrast injection [14]. Therefore, diffuse edema assessment might have to follow a different approach, such as water content quantification instead of reduced density.

Although it is surely very important to confirm the physiopathology of these HypoArt–HyperLE lesions, the notion that most myocarditis lesions are hypodense on the arterial phase is consequential by itself. Indeed, the main differential diagnosis of myocarditis is myocardial infarction, which is expected to result in a hypoperfused, hypodense area on first-pass perfusion imaging. Consequently, our study raises awareness about the fact that a hypodense area on the arterial phase is not necessarily pathognomonic of infarction, so that also its characteristics, such as the localization in the myocardium, need to be taken into account. These considerations are timely in these years of clinical implementation of photon-counting CT technology. Several studies have shown that with these systems, coronary artery stenosis and stents can be better assessed thanks to the improved spatial resolution and artifact reduction [21, 22]. Furthermore, spectral images can be reconstructed, allowing for analysis of the myocardium [23, 24]. Most likely, the possibility to combine all these features will result in broader indications for cardiac CT, including for patients with acute chest pain of cardiac origin, such as the ones we investigated in the current study.

To be noticed  that HypoArt–HyperLE lesions showed slightly higher attenuation values in the LE phase in patients without edema, while the NorArt–HyperLE lesions exhibited higher density in patients with edema as compared to those without. This occurrence might be linked to the different physiopathology of edema and myocarditis lesions in these patients. Indeed, edema in AM can be due to different phenomena, including increased free water content, but also necrosis [25, 26].

In our investigation, we noted that a majority of the HypoArt–NorLE lesions during the arterial phase were situated within the interventricular septum. This is not the typical location of myocarditis lesions that are found preferentially in the inferior and lateral walls [27], as observed also in the present study for the other types of lesions. Therefore, these areas might be attributed to beam-hardening artifacts that are prevalent in this specific region because of the high iodine concentration in the two ventricles, as already speculated [14].

There are important limitations to our study. The first limitation is the relatively low number of patients we examined. This included the low number of patients (53%) with available T2 maps. In addition, the very low rate of complications in our population did not allow us to draw conclusions on the prognostic value of HypoArt–HyperLE lesions. Comparisons between subgroups of patients were not possible for the same reason. Furthermore, there was no notable distinction in terms of density between the HypoArt–HyperLE and HypoArt–NorLE lesions during the arterial phase. Although for the abovementioned reasons we considered that HypoArt–NorLE lesions were artifacts, we do not have histologic proof of this. In addition, the CT system that was employed has a coverage of 4 cm, meaning that the myocardium cannot be imaged in a single rotation. Nevertheless, previous studies with the same system did not find systematic differences in the first-pass perfusion values of the myocardium that might be linked to this technical limitation [14].

In conclusion, most lesions of myocarditis are visible on both the arterial and the late enhancement acquisitions on spectral CT. These lesions appear more pronounced in the arterial phase in patients with myocardial edema as detected on MRI. Therefore, the utilization of the arterial phase with spectral CT helps move one further step in the designation of spectral cardiac CT as a real alternative to cardiac MRI for the diagnosis of AM.

## Data Availability

Data generated or analyzed during the study are available from the corresponding author on reasonable request.

## Abbreviations

AHA: American Heart Association
AM: Acute myocarditis
CT: Computed tomography
HypoArt–HyperLE: Hypodense at arterial phase and hyperdense at late enhancement phase
HypoArt–NorLE: Hypodense at arterial phase and isodense at late enhancement phase
LE: Late enhancement
LGE: Late gadolinium enhancement
LIE: Late iodine enhancement
MRI: Magnetic Resonance Imaging
NorArt–HyperLE: Isodense at arterial phase and hyperdense at late enhancement phase
RM: Remote myocardium
